# Stratified cities, unequal education: social class, segregation, and educational trajectories in metropolitan Bogotá

**DOI:** 10.3389/fsoc.2026.1746068

**Published:** 2026-03-17

**Authors:** Carlos Alberto Reverón Peña

**Affiliations:** Maestría en Economía y Política de la Educación (Facultad de Economía – Facultad de Ciencias de la Educación), Universidad Externado de Colombia, Bogotá, Colombia

**Keywords:** Colombia, educational inequality, educational trajectories, Latin America, residential segregation, school segregation, social class, social stratification

## Abstract

**Introduction:**

Education in Latin America operates within deeply stratified socio-spatial contexts. Despite extensive research on educational inequality, a critical gap persists in understanding how multidimensional social class - structured through economic, cultural, and social capital - interacts with residential and school segregation to produce differentiated educational trajectories across the full schooling cycle. Drawing on Bourdieusian field theory, this study examines metropolitan Bogotá as a paradigmatic case of a highly stratified Latin American metropolis in which class and territory jointly shape life-course educational opportunities. The study tests two hypotheses: (H1) individuals from lower social classes have lower probabilities of accessing, persisting in, and completing education; and (H2) living in residentially and school-segregated contexts of concentrated socioeconomic disadvantage independently reduce these probabilities.

**Methods:**

The study integrates three large-scale official datasets: the 2018 National Population and Housing Census (*n* ≈ 3 million), SABER 11 standardized tests (2010–2022; *n* ≈ 1.5 million graduates), and the 2021 Multipurpose Survey (292.281 people, of whom 87.183 are aged 3-24). A multidimensional class scheme was constructed through dimension-reduction techniques (PCA, MCA, and CATPCA) and k-means clustering. Residential and school segregation were measured across six complementary dimensions at multiple spatial scales and summarized into synthetic PCA indices. Educational trajectories across five transitions - from preschool access to high school graduation - were modeled through multilevel logistic regressions to distinguish individual capital effects from contextual territorial mechanisms.

**Results:**

Six empirically distinct and hierarchically ordered social classes were identified: Elite (0.2%), Upwardly Mobile Upper Class (2.6%), Established Middle Class (33.5%), Emerging Middle Class (6.5%), Consolidated Working Class (55.2%), and Precariat (2.0%). While nearly all Elite students complete upper secondary education, only 75% of those in the Precariat do so. Embodied cultural capital emerges as the strongest determinant of progression in primary education (OR = 4.18), with effects varying by stage - institutionalized cultural capital (parental education) gains salience at graduation (OR = 1.61). Territorial segregation independently amplifies inequalities: Precariat membership reduces graduation probability by 96% compared to the Elite, even after controlling for individual resources. Intraclass correlation coefficients indicate that territory accounts for between 6% and 23% of variance across educational transitions, peaking at secondary level.

**Discussion:**

Findings empirically substantiate Bourdieusian class theory in a Latin American context while extending it by demonstrating that the three forms of capital operate through stage-specific mechanisms rather than uniformly across the educational trajectory. Residential and school segregation operate as mechanisms of social closure that exceed individual-level disadvantages. A pattern of “double segregation” - wherein school segregation surpasses residential segregation among elite groups - reflects deliberate strategies of social distance maintenance through school choice. Universalist educational policies that overlook this territorialized class structure have limited transformative potential; structural measures simultaneously addressing territorial planning, public education strengthening, and social integration are required. The mechanisms identified are likely present in other highly unequal Latin American metropolises, with significant implications for educational policy design across Latin America.

## 1 Introduction

### 1.1 Problem contextualization

Education constitutes a central field for the analysis of inequality in contemporary societies. Educational trajectories do not operate on neutral terrain but are structured by the differential distribution of economic, cultural, and social capital across social classes. Education is, using Roemer (1998) metaphor, an “*uneven playing field*” in which children from upper classes have privileged access to the most advantaged trajectories, while those from working classes face constrained paths. In Latin America, high socio-spatial stratification intensifies these inequalities, as residential and school segregation compound to structure class-differentiated educational trajectories.

In Colombia, characterized by high levels of income and wealth inequality (Piketty, 2015), educational gaps constitute a determinant factor in social reproduction (Cárdenas et al., 2021). In metropolitan Bogotá, residential and school segregation (Mayorga and Ortiz, 2020; Murillo et al., 2018; Murillo and Carrillo-Luna, 2021; Reverón, 2024) complexify this reproduction, configuring differentiated educational trajectories wherein social class position, territory, and the properties of educational institutions intersect.

Although Colombia has advanced in understanding educational trajectories, existing research remains primarily descriptive, leaving a gap in analyses centered on the mechanisms through which class inequalities, the educational field, and urban segregation articulate to shape educational paths. This article addresses this gap by examining how the volume and composition of families' economic, cultural, and social capital, together with residential and school segregation, structure school trajectories in metropolitan Bogotá, understood as the socio-spatial configuration comprising the city and its 21 adjoining municipalities, which concentrate nearly 30% of GDP and 19% of the Colombian population.

### 1.2 Social classes, segregation and educational trajectories

Building on Bourdieu (2000) and contemporary operationalizations of his framework (Savage et al., 2013; Savage, 2015; Bennett et al., 2008; Connelly et al., 2020), this study examines how economic, cultural, and social capital -understood as accumulative and transferable resources (Wacquant, 1998)- are unequally distributed across social space, thereby structuring differentiated probabilities of access to educational opportunities. In line with the approach developed by Savage and colleagues, social classes are conceived not as reified entities endowed with autonomous collective agency, but as empirical groupings of individuals and families occupying homologous positions within social space, defined by the volume and composition of their capitals. From this perspective, economic capital conditions access to educational resources and advantageous residential locations; cultural capital operates through embodied dispositions, family educational investments, and differential knowledge of the educational system; and social capital facilitates unequal access to information, support networks, and educationally relevant opportunities. The appropriation of education is mediated by habitus, as families and students act in accordance with internalized dispositions coherent with their class-based trajectories (Bourdieu and Passeron, 1995; Ball, 2003; Lareau, 1987, 2011).

These individual and family capitals interact dynamically with specific spatial contexts. Within the educational field (Bourdieu, 2009), social class inequalities materialize in school segregation that distributes students across hierarchically differentiated educational circuits (Bonal and Bellei, 2019). Simultaneously, residential segregation produces internally homogeneous but hierarchically unequal neighborhoods (Sabatini, 2006; Wacquant, 2023). Together, these processes configure educational trajectories understood as life-course phenomena wherein gaps emerge between normative trajectories and those actually traversed, particularly across social groups (Terigi, 2009a; Sautu et al., 2020).

Beyond theoretical debates on social stratification, empirical research across multiple traditions converges in demonstrating that family socioeconomic position - whether conceptualized through occupational class schemas (Goldthorpe, 1993), composite socioeconomic status indices (Coleman, 1966), or capital volumes (Bourdieu, 2000) - constitutes the primary determinant of educational trajectories and attainment (Crawford et al., 2016; OECD, 2019). Regardless of operationalization, students from socioeconomically disadvantaged families systematically experience worse trajectories- characterized by delayed progression, elevated attrition, and diminished ultimate attainment- relative to their advantaged counterparts (Crawford et al., 2016). In Latin America, this association is particularly pronounced, reflecting the region's persistently high and spatially structured levels of inequality (Terigi, 2009a,b; Blanco et al., 2014; Cardozo, 2016).

However, social class effects on educational trajectories do not operate exclusively through direct family resource transmission. Contemporary scholarship demonstrates that residential and school segregation - the spatial concentration of families and students with similar socioeconomic positions-amplify class inequalities through four specific spatial mechanisms (Bonal and Bellei, 2019; Levy et al., 2019; Nel·lo, 2021).

- First, peer composition effects: the concentration of socioeconomically disadvantaged students tends to generate peer environments that limit academic expectations, restrict access to achievement-oriented role models, and constrain the formation of educationally beneficial social networks (Bellei, 2013; Sacerdote, 2011; Reardon and Owens, 2014). Recent research on school segregation by socioeconomic status (Owens, 2018; Palardy, 2013) have deepened understanding of these peer effects through systematic measurements from standardized tests such as PISA (OECD, 2019) and TERCE (Murillo, 2016; Krüger, 2019). Research demonstrates that students attending highly segregated, high-poverty schools consistently achieve lower academic outcomes and experience less favorable long-term trajectories, with segregation showing strong negative relationships with college success, long-term employment, and income prospects (Frankenberg et al., 2019; Ayscue et al., 2017).
- Second, differentiated institutional resources: schools serving concentrations of low-income students face systematic resource limitations, including deteriorated infrastructure, insufficient libraries and laboratories and a less experienced teaching workforce, which compound existing educational disadvantages (Mordechay and Orfield, 2017). These institutional gaps prove particularly acute in metropolitan areas where race and class disparities compound across residential and school settings. In Latin American contexts, these resource differentials intensify due to historically unequal patterns of educational investment and limited fiscal capacity to equalize school funding across socioeconomic zones (Reimers, 2000).
- Third, educational field segmentation: in quasi-market contexts, socioeconomic segregation intensifies through middle-class flight from public schools and strategic family decisions regarding school choice (Brama, 2006; Bonal and Bellei, 2019). Contemporary literature has evidenced how family strategies of school choice (Gomà and Muñoz, 2021) and the spatial distribution of educational establishments interact to affect educational attainment according to social class, producing hierarchically segmented educational circuits within the educational field with systematic quality differences along class lines (Bourdieu, 2009, 2020; Valenzuela et al., 2013). Research shows both racial and socioeconomic diversity benefit students, particularly disadvantaged ones, when within-school practices ensure genuinely equitable access to educational resources and opportunities (Ayscue et al., 2017).
- Fourth, neighborhood effects beyond school: Building on foundational work (Wilson, 1987; Jencks and Mayer, 1990), research has consolidated understanding of how segregated low-income neighborhoods systematically lack community resources-libraries, recreational centers, extracurricular programs-that complement formal schooling (Nieuwenhuis and Hooimeijer, 2016). Growing up in poor neighborhoods negatively affects school trajectories through reduced exposure to educational role models, limited access to information about educational opportunities, and constrained social networks (Leventhal and Brooks-Gunn, 2000; Wodtke et al., 2011; Chetty et al., 2016; Nel·lo, 2021), effects particularly consequential in metropolitan contexts experiencing demographic transformation without adequate policy responses (Mordechay and Orfield, 2017).

These mechanisms operate cumulatively and interactively, producing compound disadvantages that explain why segregation systematically depresses educational achievement even after controlling for family characteristics. In Latin America, characterized by exceptional socioeconomic inequality and urban segregation (Sabatini, 2006), these mechanisms acquire particular intensity, generating territorial and educational stratification that reproduces social inequality across generations (García and Fergusson, 2021; Valenzuela et al., 2013).

In Colombia, recent studies have documented that the Colombian educational system reproduces an “educational apartheid” by social class (García and Fergusson, 2021; Cárdenas et al., 2021), evidencing the strong socioeconomic segmentation of the educational field. For Bogotá specifically, research identifies high levels of school segregation (Murillo and Carrillo-Luna, 2021) and its articulation with residential segregation and social classes (Reverón, 2024; Alfonso et al., 2024), although a significant gap persists in measuring these phenomena across the complete educational trajectory.

### 1.3 Objectives, hypotheses, and research questions

This article studies the relationship between social classes, urban and school segregation, and educational trajectories in Bogotá and its metropolitan area. Specifically, the research question addresses:

*How do the economic, cultural, and social capital of individuals and their families, as well as the conditions of residential and school segregation of the areas where they live, affect access, persistence, and completion of preschool, basic, and upper secondary education?*

The main hypotheses being tested are:

H1 (Capitals and social class effects): Individuals from lower social classes -defined by lower volumes of family-level economic, cultural, and social capital-have lower probabilities of accessing preschool, persisting through primary and secondary education, and completing high school graduation.

H2 (Spatial segregation effects): Individuals living in residentially segregated areas with concentrated socioeconomic disadvantage and a school supply exhibiting high socioeconomic segregation have lower probabilities of accessing preschool, persisting through primary and secondary education, and completing high school graduation.

## 2 Materials and methods

### 2.1 Data

The research integrated diverse census, sample, and administrative information sources that, together, allow analyzing the dynamics of social class, segregation, and educational trajectories in the metropolitan area of Bogotá. The Bogotá-Cundinamarca 2021 Multipurpose Survey (Departamento Administrativo Nacional de Estadística (DANE) and Secretaría Distrital de Planeación (SDP), 2021) constitutes the main source for the construction of social classes and the estimation of statistical models. The survey offers territorially representative information on living conditions, with a sample of 292,281 people, of whom 87,183 are between 3 and 24 years old and represent approximately 3.7 million inhabitants in the metropolitan region. Census microdata from the 2018 National Population and Housing Census (DANE) were used to replicate the class stratification methodology and generate residential segregation indicators across spatial scales, supported by geostatistical cartography. Educational segregation was estimated using individual SABER 11 test results (2010–2022; ~1.5 million graduates), complemented with administrative records from SIMAT, the Unified Directory of Establishments (DUE), and DANE's formal education statistics (C600).

### 2.2 Dependent variable

Educational trajectories are analyzed across five transitions: preschool access (Kindergarten), primary persistence (grades 1–5), lower secondary (grades 6–9), upper secondary (grades 10–11), and graduation. The dependent variable operationalizes adherence to expected trajectories, defined as age-grade progression within Colombia's institutional standards. An “expected trajectory” assumes one-grade-per-year advancement within a 2-year tolerance: preschool at ages 5–7, first grade at 6–8, consecutively through eleventh grade at 16–18. Students progressing within these parameters are coded *Y* = 1; those experiencing delayed entry, excess repetition, dropout, or non-enrollment are coded *Y* = 0.

### 2.3 Independent variables

#### 2.3.1 Demographic variables

The study incorporates demographic control variables consistently identified in international educational trajectory research as significant predictors (Heck and Mahoe, 2006; Crawford et al., 2016): sex was operationalized as a dichotomous nominal variable (Male/Female). Similarly, belonging to any vulnerable population, which groups different vulnerability conditions such as cognitive, physical, or sensory disability (according to the Washington methodology), belonging to ethnic groups -shown to affect school dropout in secondary education (Heck and Mahoe, 2006), victims of violence, or rurality -consistently associated with incomplete educational trajectories; the number of people in the household as a numerical variable -reflecting resource competition within families-, and living with both parents, which distinguishes family structure through a dichotomous nominal measure -a protective factor widely documented in the literature (Crawford et al., 2016).

#### 2.3.2 Variables related to social class

Following Bourdieu (2000) multidimensional conceptualization of capital as operationalized in contemporary class analysis (Savage et al., 2005, 2013; Bennett et al., 2008; Savage, 2015), this study measures the economic, cultural, and social capital of individuals and their families through ten synthetic indices constructed to capture these three capital forms.

##### 2.3.2.1 Economic capital

Economic capital comprises material resources - income, durable goods, housing conditions, and perceived economic situation- that condition access to educational opportunities and residential locations. These indicators have been recognized as determinants of educational trajectories across multiple contexts (Blanden and Gregg, 2004; Magnuson and Waldfogel, 2016).

##### 2.3.2.2 Cultural capital

Cultural capital included three dimensions aligned with Bourdieu's tripartite distinction: institutionalized capital (educational credentials of parents and household members), embodied capital (cultural practices including reading, arts engagement, technological-cultural consumption, attendance at cultural events), and objectified (technology tool). Additionally, universal household literacy. Research documents the positive correlation between cultural capital and educational attainment and its systematic variation across social classes (Lareau, 2011; Andersen and Jæger, 2015).

##### 2.3.2.3 Social capital

Social capital is conceptualized as resources embedded in and mobilizable through social relationships structured by occupational position, residential networks, and associational participation. From a relational perspective, it is unequally distributed across class positions, as social networks themselves are class-structured and reproduce social advantage (Espinoza, 1999; Savage et al., 2013). It is operationalized through three dimensions: parents' occupational status as a proxy for access to class-differentiated networks (Goldthorpe, 1993); residential rootedness, measured as years in the current dwelling and capturing local network embeddedness (Levy et al., 2019; Bonal et al., 2019); and organizational and community ties, measured through associational membership, neighborhood satisfaction, and perceived support from neighbors and friends.

Supplementary Table 1 summarizes the ten measurement variables operationalizing.

#### 2.3.3 Territorial variables of the educational field

In addition to social class, variables were incorporated that capture structural characteristics of the educational field in residential areas (neighborhood clusters) informed by research on residential segregation and educational opportunity structures (Lareau, 2011; Van Ham et al., 2021): percentage of provision with full-day schooling -whose differential effects across educational levels have been documented (Cooper et al., 2010), public-private composition of enrollment -central to quasi-market segregation dynamics and middle-class flight from public schools (Brama, 2006), territorial accessibility (average home-school commute time), academic quality (standardized tests), and school failure (dropout and grade repetition) -reflecting institutional capacity and neighborhood educational climate. These variables allow examining how territorial conditions of the educational field differentially configure educational opportunities as suggested by field theory applied to education (Bourdieu, 2009).

#### 2.3.4 School and residential segregation variables

Residential and school segregation is inherently multidimensional, and different measures can exhibit contradictory directional trends (Massey and Denton, 1988; Orfield et al., 2014; Musterd, 2020; Reardon and Owens, 2014; Mordechay and Terbeck, 2024). This multidimensional approach is particularly relevant because different dimensions of segregation can operate through distinct mechanisms at different moments in the educational trajectory. Additionally, as documented by Orfield et al. (2014) and Mordechay and Terbeck (2024), different segregation measures can exhibit contradictory trends: analyses relying on a single global measure might erroneously conclude about the direction or magnitude of segregation's educational effects.

Within this multidimensional framework, and following Massey and Denton (1988), this research measures six complementary dimensions of segregation: evenness/unevenness, exposure/interaction, concentration/clustering, centralization, spatial clustering, and spatial autocorrelation, with the purpose of incorporating diverse methodological approaches given the complex and complementary nature of segregation (Allen and Vignoles, 2007). Spatial autocorrelation was evaluated through the Global Moran Index and Local Indicators of Spatial Association – LISA – (Anselin, 1995), to identify territorial clustering patterns fundamental for understanding spatial inequality patterns (Atkinson and Kintrea, 2001). The indicators were calculated at multiple spatial scales to capture both macro and micro-segregation processes (Wilson, 1987; Jencks and Mayer, 1990).

Supplementary Table 2 summarizes the six segregation dimensions, their definitions, indicators and associated mechanisms affecting educational trajectories. Descriptive statistics are reported in Supplementary Table 3.

### 2.4 Analytic methods

The analysis of educational trajectories follows a sequential transition approach that models the probability of progressing across key educational stages. Following Mare (1980) and subsequent refinements (Fernández and Martínez, 2016; Bi and Liu, 2024), this study estimates separate logistic regression models for each educational transition, rather than imposing a single continuous process over the entire trajectory. This modeling choice offers two main methodological advantages: First, it yields transition-specific probabilities that are analytically independent across stages. Rather than assuming that the same set of mechanisms governs the entire educational process, stage-specific logistic models allow the determinants of access, persistence, and completion to be examined as distinct, though related, social processes. Second, this approach captures how social and territorial effects vary across the educational life course, tracing how class-based advantages and territorial inequalities cumulate, attenuate, or transform along the educational trajectory.

Accordingly, separate logistic regressions were estimated for each stage of the trajectory – access to preschool, on-time progression through primary, lower and upper secondary, and high school completion. The dependent variable operationalizes educational trajectory adherence relative to Colombia's normative age-grade progression standards. Following Ministry of Education, an “expected trajectory” assumes students advance through the system within a two-year tolerance window around the modal age for each grade. For each educational stage, *Y* is coded as a binary variable:

- Preschool access: *Y* = 1 if enrolled at age 5–7; Y = 0 otherwise
- Primary persistence (grades 1–5): *Y* = 1 if enrolled in age-appropriate grade (ages 6–12, ±2 years); *Y* = 0 if not enrolled, dropped out, or over-age beyond tolerance
- Lower secondary persistence (grades 6–9): *Y* = 1 if on-track (ages 11–16, ±2 years); *Y* = 0 otherwise
- Upper secondary persistence (grades 10–11): *Y* = 1 if on-track (ages 15–18, ±2 years); *Y* = 0 otherwise
- High school completion: Y = 1 if graduated by ages 18–24; Y = 0 if not completed, dropped out, or never enrolled

This binary operationalization treats educational trajectories as a sequence of “at risk” transitions where students either progress as expected (*Y* = 1) or experience disruption through non-enrollment, dropout, or excessive grade retention (*Y* = 0). The 2-year tolerance accommodates normal variations in school entry age and grade repetition, while still identifying educationally vulnerable populations. The transition probability is modeled as:


$$\begin{array}{l} \mathrm{Pr}\left(Y_{i}=1|X\right)=F\left(\beta_{0}+\beta_{1}X_{1i}+\cdots +\beta_{p}X_{pi}\right),F\left(x\right)=\frac{e^{x}}{1+e^{x}}. \end{array}$$

β_0_ is the intercept, and β_1_, β_2_, ..., β_*p*_ are the parameters associated with the set of independent variables *X*_1_, *X*_2_, ..., *X*_*p*_ that represent class conditions, segregation, and educational field characteristics. Odds ratios ($e^{\beta_{j}}$) are reported to facilitate interpretation of effect sizes.

#### 2.4.1 Multilevel modeling

Considering the nested structure of the data -where individuals are grouped into territorial units - multilevel logistic regressions were estimated. The choice of multilevel models (Raudenbush and Bryk, 2002) responds to the consolidation in international literature of the recognition that educational data has a hierarchical structure. Since the seminal works of Wilson (1987) on “neighborhood effects” and Jencks and Mayer (1990) on their operating mechanisms, educational research has established that school trajectories are conditioned by both individual characteristics and territorial context. Multilevel models allow for the decomposition of this variance (Duncan et al., 1997; Palardy, 2013), distinguishing composition effects from genuine contextual effects of territory.

The general random-intercept specification is:


$$\begin{array}{l} \log\left[\frac{P(Y_{ij}=1)}{1-P(Y_{ij}=1)}\right]=\beta_{0j}+\sum_{k=1}^{n}\beta_{1j}X_{kij}+\varepsilon_{ij}\\ \;\beta_{0j}=\gamma_{00}+\sum_{k=1}^{n}\gamma_{01}Z_{mj}+\mu_{0j}\\ \;\beta_{1j}=\gamma_{10}\\ \;\log[\mathrm{Pr}(Y_{ij}=1)]=\gamma_{00}\;+\;\gamma_{10}X_{kij}+\gamma_{01}Z_{mj}+\mu_{0j}+\varepsilon_{ij} \end{array}$$

where *X*_*kij*_ and *Z*_*mj*_ denote individual-level and contextual predictors, respectively, and μ_0*j*_ captures unobserved territorial heterogeneity. The Intraclass Correlation Coefficient (ICC) quantifies the proportion of variance attributable to territory differences. Marginal *R*^2^ and conditional *R*^2^ following Nakagawa and Schielzeth (2013) providing a comprehensive assessment of model fit and the relative importance of compositional vs. contextual determinants.

This approach fulfills three key analytical functions: it ensures correct statistical inference by accounting for clustered observations; it enables inequality decomposition by distinguishing compositional from contextual territorial effects, a central distinction for assessing whether residential segregation generates additional inequality (Van Ham et al., 2021; Lareau and Goyette, 2014); and it quantifies territorial stratification through the Intraclass Correlation Coefficient (ICC), operationalizing neighborhood effects as the share of variance attributable to between-territory differences (Chetty et al., 2016; Nel·lo, 2021). Given the nested data structure (individuals within territorial units), multilevel logistic regressions constitute the primary analytical approach. Standard logistic models are reported in Supplementary material as robustness checks, showing consistent direction and significance of effects albeit with less conservative standard errors.

#### 2.4.2 Multivariate statistical analysis

To construct the independent variables related to the different forms of capital (cultural, economic, and social), dimension reduction techniques appropriate to variable measurement levels were employed: principal Component Analysis (PCA) for continuous variables and Multiple Correspondence Analysis (MCA) for categorical variables -a method that represents associations among categories through decomposition of chi-square distances. For mixed-type variables, Categorical Principal Component Analysis (CATPCA) with optimal scaling was used. This procedure yielded eight capital indices; two variables were included directly without dimensional reduction: household per capita income and household literacy. Together, these ten variables operationalize the three forms of capital employed as independent variables in regression models. Quality metrics for the constructed indices -including explained variance/inertia and Cronbach's alpha coefficients - are in Supplementary Table 4.

Additionally, these synthetic capital indices were used to construct empirical social class groupings through cluster analysis. Using standardized synthetic indices of each capital and relying on the first three principal components derived from each dimension (retaining 91.8, 100, and 100% of variance for economic, cultural, and social capital respectively), k-means cluster analyses were performed to identify distinct social class groupings. Cluster stability was evaluated through discriminant analysis, assessing group separation using the F-test and the proportion of variance explained. The optimal number of clusters was selected by minimizing Wilks' Lambda, examining the magnitude of the canonical correlations associated with each discriminant function, and applying Box's M statistic to test the homogeneity of covariance matrices across groups. Model robustness was further assessed through complementary multinomial logistic regressions.

##### 2.4.2.1 Several methodological considerations merit acknowledgment

First, k-means clustering exhibits sensitivity to the pre-specified number of groups; the selection of six classes was justified both empirically (Wilks' Lambda ≈ 0) and aligns conceptually with established multidimensional class frameworks (Savage, 2015). Second, while discriminant functions and multinomial models confirm classification validity, potential misclassification remains at boundaries between adjacent classes, particularly between emergent middle and consolidated working classes. Third, the use of three principal components per capital dimension balances information retention against the risk of overfitting; this approach preserved substantially more variance than single-component solutions while maintaining computational tractability for the clustering algorithm. Finally, temporal stability warrants consideration given the 2021 data collection period (post-pandemic recovery); replication of the synthetic index construction procedure with 2018 Census data yielded strong consistency in territorial socioeconomic (correlation = 0.955 at territorial unit level), supporting the stability of underlying multidimensional inequality patterns, though the specific class boundaries and optimal cluster number derive from the 2021 calibration sample.

#### 2.4.3 Construction of synthetic segregation indices

Although all segregation indicators described above were calculated using specialized programs (Geosegregation Analyzer, R, STATA), in the final logistic regression models one indicator per dimension was prioritized due to the need to avoid multicollinearity: Theil Entropy Diversity Index (evenness/unevenness), Isolation Index (exposure/interaction), Location Coefficient (concentration/clustering), and Local Moran Index – LISA (spatial autocorrelation). These four indicators were selected for their technical characteristics that avoid distortions related to differences in population sizes of social classes across different UPZs or schools.^1^

From these four normalized indicators, Principal Component Analysis (PCA) was applied to construct synthetic segregation indices. The decision to apply for PCA responds to three core methodological challenges. First, structural multicollinearity: simultaneous inclusion of the original 12 segregation variables (one residential and one school-based for each of the six social classes) in regression models generated severe multicollinearity problems (VIF>5), compromising the stability of estimated coefficients. Second, heterogeneity in measurement scales complicates interpreting regression coefficients (Reardon and Firebaugh, 2002). Third, the parsimony-comprehensiveness dilemma: capturing multidimensionality requires multiple indicators, but their simultaneous inclusion generates overfitting.

Separate PCAs were performed for residential and school segregation of each of the six identified social classes, extracting the first component. Resulting components retain between 47.8% and 87.1% of original variance. This dimensional reduction transforms four original dimensions (evenness/Theil, exposure/isolation, concentration/location quotient, and clustering/Moran) into a single component per class capturing the general segregation gradient. Subsequently, to address multicollinearity in regression models, adjacent classes were aggregated. This reduced the original 12 segregation variables to synthetic indices for four final groupings: elite, upwardly mobile upper class, aggregate middle class, and aggregate working class. This aggregation improved model fit (AIC and BIC indicators) while enabling simultaneous capture of multiple segregation dimensions. Tables in Supplementary material present communalities and factor loadings for each component index by social class.

All synthetic indices were standardized (mean = 0, SD = 1) to facilitate substantive interpretation: reported odds ratios correspond to changes in educational transition probabilities associated with a one-standard-deviation increase in segregation level. Since principal components integrate multiple dimensions (evenness, exposure, concentration, clustering), one standard deviation represents a substantive change in the overall pattern of territorial segregation. In the reported results (Table 4), odds ratios should be interpreted as the multiplicative change in the odds of completing each educational transition given increases in territorial segregation patterns experienced by each social class.

## 3 Results

### 3.1 Social classes: an empirical typology for metropolitan Bogotá

In Colombia, unlike in most Latin American or global research contexts, studies on social class remain scarce and have largely focused on income or occupational status. Addressing this gap, this study develops a multidimensional and relational classification of social classes in metropolitan Bogotá, grounded in Bourdieu's theory of capitals. The analysis yielded six statistically distinct and hierarchically ordered social classes – Elite, Upwardly Mobile Upper Class, Established Middle Class, Emerging Middle Class, Consolidated Working Class, and Precariat – reflecting the deep stratification and persistent inequalities that structure Bogotá's urban society (Table 1).

**Table 1:** Social class structure in metropolitan Bogotá: demographic composition and capital distribution.

**Capital**	**Indicator**	**Elite (0.2%)**	**Upward upper (2.6%)**	**Established middle (33.5%)**	**Emerging middle (6.5%)**	**Consolidated working (55.2%)**	**Precariat (2.0%)**
Economic capital	Per capita income (USD/month)	$4,220	$1,487	$313	$249	$116	$107
Economic capital	Monetary poverty	0%	0%	15%	30%	59%	65%
Economic capital	Multidimensional poverty	0%	0%	0%	4%	11%	61%
Economic capital	Computer ownership	98%	98%	91%	75%	41%	39%
Cultural capital	Mother's education: university+	88%	87%	42%	32%	7%	10%
Cultural capital	Father's education: university+	86%	85%	40%	26%	3%	6%
Cultural capital	Family schooling (years)	17.4	16.9	13.6	11.9	9.5	5.9
Cultural capital	Children: read books (leisure time)	48%	37%	23%	26%	9%	9%
Cultural capital	Children: zero hours sports/week	20%	28%	38%	31%	57%	60%
Social capital	Two-parent household	58%	66%	58%	51%	39%	35%
Social capital	Head: directors/professionals	63%	63%	35%	29%	10%	15%
Social capital	Head: laborers/sales/elementary	16%	15%	36%	50%	75%	69%
Social capital	Residential rootedness Bogotá	85%	87%	84%	78%	74%	60%
Other	Vulnerable population	6%	7%	5%	12%	10%	14%

(a) USD conversion using April 2021 average rate: 3,750 COP/USD. (b) At least one parent with completed university or graduate education. (c) Includes directors/managers + professionals/scientists. (d) Includes craft/operative/artisan + service/sales + elementary workers.

Source: Author's calculations using EM-2021 and CNPV-2018 (DANE).

The Elite Class (0.2%) concentrates exceptional capital levels: monthly per capita incomes around USD 4,220, full higher education among adults, and almost universal access to digital and cultural goods (98% own computers). Families are typically nuclear, long-established in the city (85%), and household heads are executives or professionals (63%). Children engage regularly in sports and reading and attend elite private schools.

The Upwardly Mobile Upper Class (2.6%) shows high but less consolidated capital, with per capita income near USD 1,487, 87% with higher education and professionals/managerial occupations (63%). Their children combine structured extracurricular activities with intensive educational investment, reflecting an aspirational trajectory of consolidation rather than inheritance of privilege.

The Established Middle Class (33.5%) demonstrates moderate economic security (USD 313 per capita), balanced capital distribution, and 40% parental higher education. Household heads are professionals or mid-level employees (35%), with moderate cultural participation among children.

The Emerging Middle Class (6.5%) represents upwardly mobile households with USD 249 per capita income and partial vulnerability (30% monetary poverty). Families are larger (65% with four or more members), with lower parental education (~12 years) and occupations in services/sales (50%).

The Consolidated Working Class (55.2%) -constituting most metropolitan households-survives on USD 116 per capita monthly, with 59% in monetary poverty. Heads are manual workers or vendors (75%) with below-secondary education (9.5 years). Children's cultural engagement is minimal, consistent with Lareau's (2011) “natural growth” pattern.

The Precarious Working Class (2.0%) experiences extreme deprivation: USD 107 per capita, over 60% multidimensional poverty, predominantly single-mother families (35% two-parent), and minimal educational/cultural access. Around 14% belong to vulnerable or marginalized groups (ethnic minorities, rural migrants, victims of violence, or persons with disabilities), exemplifying the cumulative disadvantages described by Standing (2011) concept of the precariat.

### 3.2 Social classes and urban space

**Figure 1:**
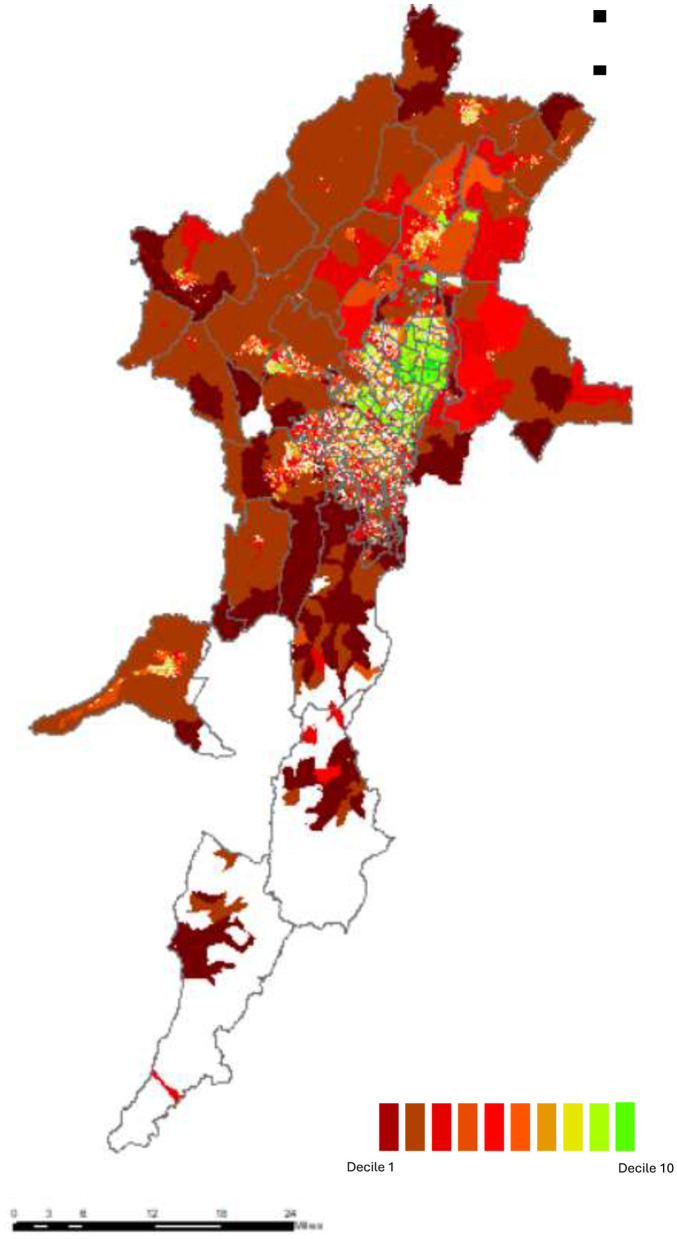
Synthetic social class index by section/neighborhood and block. Source: Author's calculations based on the methodology described and data from EM-2021 and CNPV-2018 from DANE.

The spatial distribution of social classes in metropolitan Bogotá reveals profound residential segregation patterns that materialize class hierarchies in urban space (Figure 1). Individuals from elite and upper classes concentrate in northern Bogotá, middle classes families reside predominantly in intermediate western zones, and working-class households concentrate in the southern periphery, with the precariat families dispersed across marginal micro-territories. This spatial configuration reproduces the historic north-south socioeconomic division (Aliaga and Álvarez, 2010), aligning with contemporary patterns of poverty concentration and occupational stratification (Mayorga, 2021; Bucheli, 2020; Alfonso, 2023; López and Ceballos, 2021).

Metropolitan Bogotá exhibits moderate-to-high residential segregation by social class that intensifies progressively at finer spatial scales – a pattern consistent with hierarchical urban organization characteristic of Latin American metropolises: 60% of individuals would require relocation across census blocks to achieve evenness, decreasing to 50% at neighborhood level. Segregation follows a “U-shaped” pattern: 93% redistribution needed for Elite, 40–50% for middle classes, 55–60% for working classes (Table 2). Crucially, segregation intensity follows a distinctive “U-shaped” pattern across the class spectrum, wherein extreme classes—both privileged and disadvantaged—exhibit maximum spatial isolation. Achieving residential evenness would require redistributing 93% of elite individuals across census blocks (86% across neighborhoods) and 78% of upwardly mobile upper-class individuals (71% across neighborhoods). Middle classes present moderate segregation, while working classes display intermediate levels. This U-shaped configuration evidence that spatial segregation operates not merely as income sorting but as active social boundary maintenance by elite groups seeking distinction and working-class communities forging solidarity through territorial clustering.

**Table 2:** Residential segregation indices by social class at UPZ, neighborhood/section, and census block.

**Social class**	**UPZ**				**Neighborhood/census section**				**Census block**			
	**Segreg. (ISs)**	**Entr. (H)**	**Gini (G)**	**Atkin. (0.5)**	**Segreg. (ISs)**	**Entr. (H)**	**Gini (G)**	**Atkin. (0.5)**	**Segreg. (ISs)**	**Entr. (H)**	**Gini (G)**	**Atkin. (0.5)**
Elite-upper	0.83	0.36	0.94	0.88	0.86	0.39	0.95	0.9	0.93	0.48	0.98	0.96
Upward upper	0.67	0.32	0.83	0.65	0.71	0.37	0.85	0.65	0.78	0.45	0.91	0.82
Established middle	0.28	0.1	0.42	0.14	0.53	0.29	0.69	0.41	0.62	0.39	0.78	0.56
Emerging middle	0.16	0.02	0.23	0.05	0.27	0.06	0.36	0.15	0.41	0.14	0.55	0.35
Consolidated working	0.32	0.15	0.47	0.21	0.52	0.29	0.68	0.39	0.61	0.35	0.78	0.53
Working-precariat	0.25	0.04	0.36	0.12	0.4	0.14	0.56	0.29	0.68	0.34	0.84	0.72

Source: Author's calculations using EM-2021 and CNPV-2018 (DANE).

Similarly, spatial autocorrelation analysis reveals pronounced socio-spatial clustering of class conditions, with Moran's I values increasing at finer scales. Economic capital exhibits the highest autocorrelation across all territorial levels, followed by cultural and social capital. Autocorrelation is strongest for elite and upwardly mobile fractions (high territorial concentration), while the precariat shows low block-level values (dispersion across heterogeneous micro-territories). Effects are most pronounced at the UPZ scale among privileged groups (See are reported in Supplementary material (Results are reported in Supplementary Table 1).

### 3.3 Educational field, social classes, and school segregation

The educational field in metropolitan Bogotá (1.6 million students: 62% public, 38% private) exhibits three axes of inequality that consolidate class-differentiated circuits. First, the public-private distinction constitutes a central axis of social differentiation: 100% of the Elite attend private schools, compared to 27% of the Precariat. Second, segmentation by academic quality: achievement gaps on SABER 11 standardized tests between Elite and Precariat reach 116.5 points in overall scores, with greater inequality in English (37.7 points) and lesser disparity in critical reading (18.3 points). Third, differential access to exclusive resources: 100% of the Elite vs. 19% of the Precariat accesses full-day schooling, and 63% vs. < 0.1% attends bilingual education, consolidating cumulative advantages in cultural and linguistic capital that reproduce social hierarchies.

These structural conditions materialize in class-differentiated school choice strategies and trajectories, consolidating segmented educational circuits that reproduce these inequalities:

Upper classes (Elite and Upwardly Mobile) concentrate almost exclusively in highly selective private schools (>99%), A+ category (>95%), with full-day schedules and elevated bilingualism (33–67%), investing ~USD $1,000/month. These patterns reflect strategies of status conservation and construction of exclusive networks (Ball, 2003; Tiramonti and Ziegler, 2008).

Middle classes pursue education as upward mobility vehicle. The Established Middle Class (33.5%) attends predominantly private schools (68%, USD $220/month) near residence (74%). The Emerging Middle Class (6.5%) distributes between public-private sectors (60%-40%) with reduced full-day access (43%), evidencing economic vulnerability while maintaining mobility aspirations (Espinoza and Barozet, 2009).

Working classes (57.2%) and Precariat (2%) adapt through “choice of the necessary” (Bourdieu, 2020), attending nearby public schools (74–88%) with limited full-day schedules (19–30%), reproducing residential segregation patterns (Palardy, 2013). Precariat shows higher enrollment in evening/flexible models (32%), reflecting interrupted trajectories.

**Figure 2:**
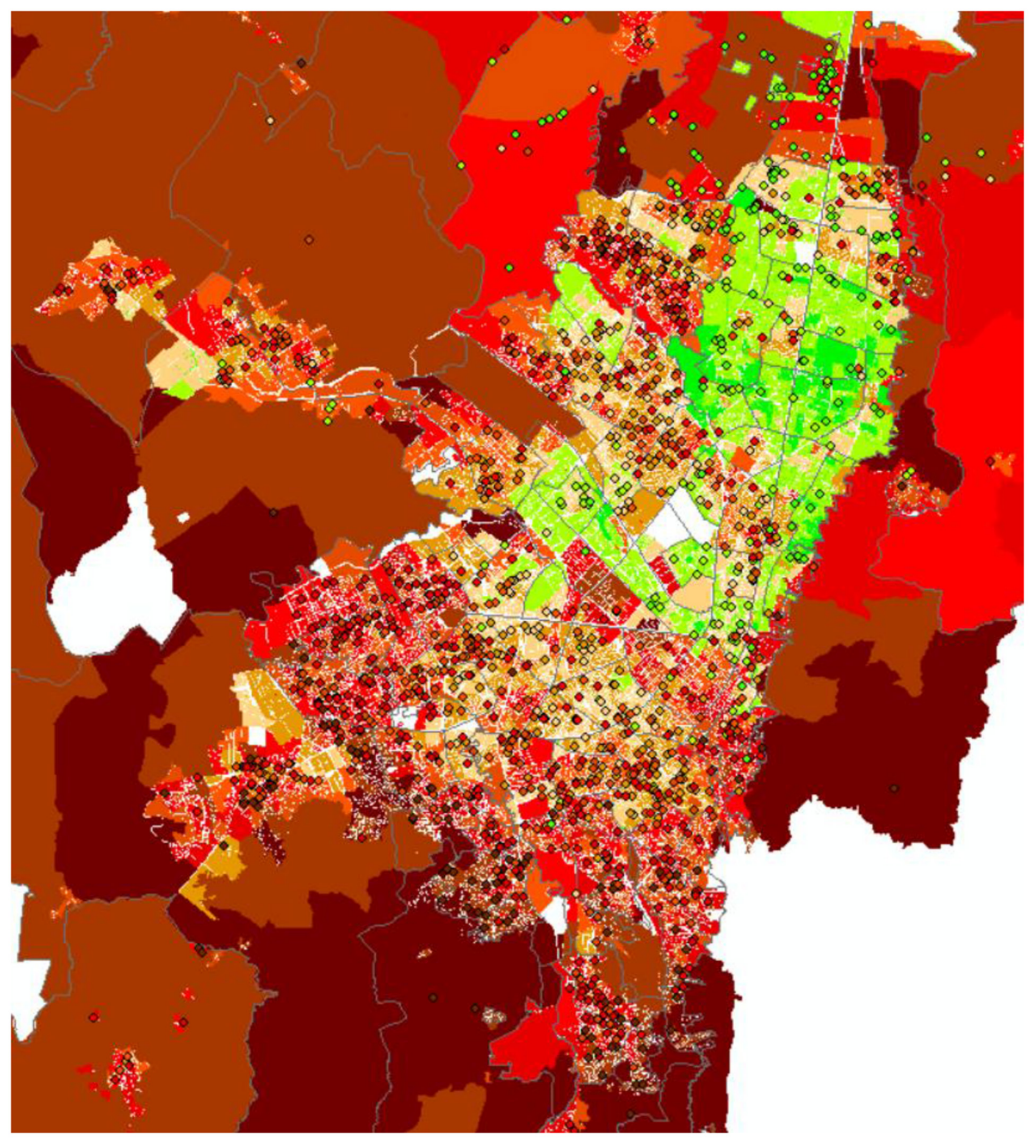
Residential and School Spatial Distribution by Social Class in Metropolitan Bogotá. Source: Author's calculations based on the methodology described and data from ICFES-SABER 11 tests, MEN, and DANE-EMB (2021).

These differentiated strategies consolidate some of Latin America's highest school segregation levels. Colombia -alongside Peru, Chile, and Mexico- exceeds regional averages (Murillo and Carrillo-Luna, 2021; Vásquez, 2016), with segregation particularly pronounced among upper socioeconomic groups. In Bogotá, school segregation interweaves with urban development, showing strong correspondence between school location and neighborhood class composition (Figure 2).

Notably, school segregation exceeds residential segregation for upper classes, the Emerging Middle Class, and the Precariat—a pattern like that observed in European countries (Van Ham et al., 2021). In contrast, for the Consolidated Working Class, residential segregation surpasses school segregation. A distinctive “U-shaped” pattern emerges: segregation reaches 93% for the Elite, moderates to 40–50% for middle and consolidated working classes, and elevates to 55% for the Precariat (Table 3). These values confirm the Elite's extreme isolation and more pronounced segregation between socially distant classes (Murillo and Carrillo-Luna, 2021).

**Table 3:** School segregation indices in metropolitan Bogotá at school level by social class.

**Social class**	**Segreg. (ISs)**	**Entr. (H)**	**Gini (G)**	**Atkin. (0.5)**
Elite-upper	0.93	0.27	0.97	0.95
Upwardly mobile upper	0.83	0.24	0.93	0.85
Established middle	0.48	0.11	0.63	0.33
Emerging middle	0.42	0.08	0.57	0.29
Consolidated working	0.41	0.08	0.57	0.28
Precariat	0.55	0.13	0.73	0.51

Source: Author's calculations based on the methodology described and data from ICFES – SABER 11.

Taken together, the educational field in metropolitan Bogotá is hierarchical, segmented by social class, and spatially segregated through three complementary processes: historic fragmentation into differentiated public/private circuits with variations in quality; class-differentiated school selection strategies; and high school segregation that reinforces residential segregation, consolidating a double segregation system structuring unequal educational opportunities.

### 3.4 A matter of survival: determinants of educational trajectories

The longitudinal analysis of educational trajectories from preschool through high school graduation demonstrates that social class operates as the most robust predictor of educational attainment in metropolitan Bogotá, systematically surpassing the explanatory power of gender, family configuration, or isolated territorial variables. The differential possession of economic, cultural, and social capital according to class position structures radically unequal probabilities of access, persistence, and completion at each successive stage of the educational trajectory—revealing education not as meritocratic selection but as class-structured sorting.

**Figure 3:**
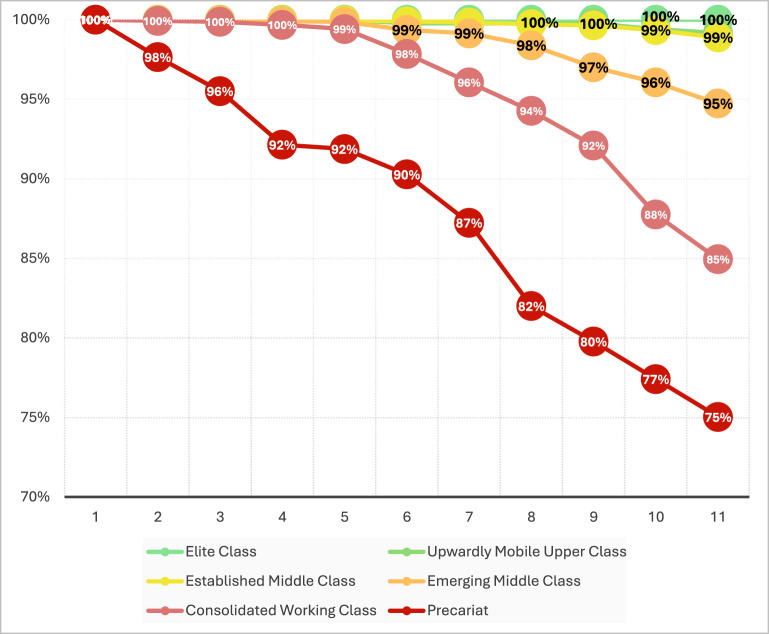
School Survival Rate of Students (age 18 to 23) Throughout the Educational Trajectory by Social Class. Source: Author's calculations based on the methodology described and data from DANE-EMB (2021).

Figure 3 presents educational survival curves that starkly illustrate this class-based stratification. Tracking cohorts who enter first grade, the divergent pathways to eleventh grade completion reveal cumulative advantage and disadvantage processes characteristic of social reproduction mechanisms. The pattern is unequivocal: of every 100 children from the Elite or Upwardly Mobile Upper Class who enter first grade, 100 complete eleventh grade—educational success approaching certainty. In sharp contrast, only 85 per 100 from the Consolidated Working Class and a mere 75 per 100 from the Precariat manage to “survive” until upper secondary completion. This 25-percentage-point gap between Elite and Precariat represents not individual failure but structural exclusion—a quarter of the most disadvantaged students systematically filtered out across the educational pipeline.

These differences between complete and incomplete trajectories, in turn, reflect inequalities in the pathways, grade repetition, and dropout patterns experienced throughout the school cycle by students from different socioeconomic backgrounds (Table 4).

**Table 4:** Odds ratios of logistic and hierarchical regression models across the educational trajectory.

**Dimension**	**Variable**	**Preschool**		**Primary**		**Secondary**		**High school**		**Complete school**	
		**OR**	**Signif**.	**OR**	**Signif**.	**OR**	**Signif**.	**OR**	**Signif**.	**OR**	**Signif**.
School and residential segregation variables	Elite class segregation	1.04		1.34	^**^	1.34	^**^	0.99		1.21	^***^
	Upper ascending class segregation	0.88	^*^	0.72	^*^	0.63	^***^	0.85	.	0.64	^***^
	Middle class (agr) segregation	0.97		0.93		1.03		1.09		1.05	
	Popular class (agr) segregation	0.89	^*^	1.01		1.09		1.01		0.97	
Educational field variables at territorial level	Bilingualism	0.98		1.06		1.04		1		1.11	^**^
	Full school day	1.09	.	1.03		0.92		0.91		1.02	
	Enrollment in public schools	0.96		1.17		1.12		1.01		0.98	
	Unavailability of school offer	1.01		1.01		1.05		0.97		0.93	.
	Low quality schools	1.11	.	1.14		1.04		1.21	^**^	1.06	
	School failure	1.03		0.79	^*^	0.83	.	0.87	^*^	0.92	.
Social class variables	Social capital - organization and trust	0.98	^***^	1		0.95	^***^	0.98	^***^	1	
	Social capital - residential roots	1.2	^***^	1.65	^***^	1.42	^***^	1.27	^***^	1.11	^***^
	Social capital - parents' occupation	1.05	^***^	1.22	^***^	1.18	^***^	0.99	^*^	1.04	^***^
	Economic capital - assets	1.17	^***^	0.93	^***^	1.22	^***^	1.34	^***^	1.47	^***^
	Economic capital - income	1.22	^***^	0.77	^***^	0.94	^***^	0.94	^***^	1.48	^***^
	Economic capital - housing	1.02	^***^	0.96	^***^	1.14	^***^	1.25	^***^	1.17	^***^
	Economic capital - perception	1.02	^***^	1.24	^***^	1.12	^***^	1.11	^***^	1.11	^***^
	Cultural capital recog. - education	1.11	^***^	1.25	^***^	1.44	^***^	1.61	^***^	1.41	^***^
	Cultural capital incorp. - practices	0.89	^***^	4.18	^***^	2.26	^***^	2.12	^***^	1.56	^***^
	Cultural capital - literacy	0.81	^***^	0.79	^***^	1.32	^***^	1.58	^***^	2.5	^***^
Individual and demographic variables	Sex	1.07	^***^	1.09	^***^	1.67	^***^	1.72	^***^	1.67	^***^
	Belonging to vulnerable population	0.89	^***^	0.75	^***^	0.8	^***^	0.95	^**^	0.85	^***^
	Lives with both parents	0.77	^***^	1.24	^***^	1.66	^***^	1.77	^***^	1.36	^***^
	Number of people in household	0.98	^***^	0.98	^***^	0.9	^***^	0.86	^***^	0.87	^***^
	**Marginal** ***R*^2^**	**0.07**		**0.43**		**0.36**		**0.4**		**0.38**	
	**Conditional** ***R*^2^**	**0.1**		**0.51**		**0.45**		**0.44**		**0.4**	
	**Delta_G**	**0.03**		**0.26**		**0.2**		**0.2**		**0.14**	
	**Confusion overall**	**0.55**		**0.94**		**0.92**		**0.8**		**0.85**	
	**ICC**	**0.06**		**0.18**		**0.23**		**0.18**		**0.16**	

Signif. codes: 0 “^***^” 0.001 “^**^” 0.01 “^*^” 0.05 “.” 0.1 “ ” 1.

Source: Own calculations based on the methodology described. Bold rows indicate overall model fit statistics for the complete model.

#### 3.4.1 Preschool access

In metropolitan Bogotá, 54% of children aged 3–5 access preschool education. The multilevel model explains 10% of variance (conditional *R*^2^), with 6% attributable to territorial differences (ICC). Family income emerges as the most determinant factor (+22%), followed by residential rootedness (+20%), durable goods possession (+17%), and household educational level (+11%). Counterintuitively, cultural practices show negative effect (-11%), attributable to the COVID-19 pandemic context when families with higher cultural capital postponed enrollment until vaccine availability. Among territorial variables, greater provision of full-day schooling in the zone increases access (+9%), reflecting preferences of families requiring extended childcare, while low school quality shows counterintuitive positive effect (+11%), explicable through coverage-quality trade-off. Living with both parents reduces access (-23%), a finding specific to the pandemic context.

#### 3.4.2 Primary education persistence

In primary education (ages 6–10), 95% maintain expected trajectory, representing the most universal level. The model explains 51% of the variance, with 18% attributable to territory, achieving 94% predictive accuracy. Embodied cultural capital (household daily practices) quadruples the probability of persistence (OR = 4.18), constituting the strongest effect across the entire educational trajectory. Residential rootedness also demonstrates robust effect (+65%), alongside household educational level (+25%) and parental occupation (+22%). Among territorial variables, Elite segregation in residential zone increases persistence probability (+34%), while local school failure reduces it (−21%).

#### 3.4.3 Secondary education persistence

In secondary education (ages 11–14), 93.1% maintain expected trajectory. The model explains 45% of variance with 23% attributable to territory (the highest ICC across the entire trajectory), achieving 92% predictive accuracy. Embodied cultural capital maintains robust effect though lower than in primary education (OR = 2.26), while household educational level increases in relevance (+44%). All capitals prove statistically significant: residential rootedness (+42%), household literacy (+32%), durable goods possession (+22%), parental occupation (+18%), housing conditions (+14%), and economic perception (+12%). Elite segregation in residential zone shows strong positive effect (+34%). Gender gaps widen substantially: females have 67% higher probability of maintaining expected trajectory, living with both parents increases probability by 66%, belonging to vulnerable populations reduces it by 20%, and each additional household member decreases it by 10%.

#### 3.4.4 Upper secondary education trajectory

In upper secondary, 84.5% maintained expected trajectory (69% still attending and 15% already graduated), while 15.5% present incomplete trajectories. The model explains 44% variance, with 18% attributable to territory. The capital configuration shows relevant reorganization: institutionalized cultural capital (family education) reaches maximum incidence (+61%), followed by household literacy (+58%), while embodied capital maintains significant effect though lower than in previous levels (OR≈2.0). Among economic capitals, durable goods possession (+34%), housing conditions (+25%), and residential rootedness (+27%) maintain positive influence. At the territorial level, three variables are significant: upper-class segregation reduces continuity probability (−15%), local low school quality increases it (+21%), and local school failure reduces it (−13%). This pattern suggests that in upper secondary, family resources compensate for territorial limitations, for instance through mobility toward higher-quality schools. Females present 72% higher probability of maintaining trajectory, living with both parents increases it by 77%, and each additional household member reduces it by 14%.

#### 3.4.5 Educational trajectory at completion and graduation

At the completion stage, 87% completed expected trajectory, 9% drop out or do not enroll, and 4% present lag. The model explains 40% of variance, with 16% attributable to territory. Cultural capitals consolidate as the most determinant factors: household literacy multiplies graduation probability by 2.5, followed by family education (+41%) and cultural practices (+56%). Among economic capitals, durable goods possession (+47%), family income (+48%), housing conditions (+17%), and economic perception (+11%) show consistent effects. Regarding social capital, residential rootedness maintains positive though more moderate effect (+11%). At the territorial level, Elite segregation in residential zone increases graduation probability (+21%), as does bilingual school presence (+11%), evidencing cumulative advantages of contexts with high educational resource concentration. In contrast, local school failure (-8%) and low pertinent educational provision (-7%) reduce completion probability. Females maintain 67% advantage, living with both parents increases probability by 36%, belonging to vulnerable populations reduces it by 15%, and each additional household member decreases it by 13%.

#### 3.4.6 Categorical social class as principal predictor

The model utilizing categorical social class (comparing all classes against Elite) confirms its determinant role, controlling for individual, family, and territorial variables. Compared to the Elite, graduation probability reduces progressively according to position in the social hierarchy: upwardly Mobile Upper Class −31%, Established Middle Class −50%, Emerging Middle Class −71%, Consolidated Working Class −87%, and Precariat −96% (Table 5). Additionally, residing in Elite concentration zone increases graduation probability by 15%, while residing in Upwardly Mobile Upper-Class zone reduces it by 16% and in working-class zone by 23%. These gaps evidence that social class not only determines the starting point, but its effect accumulates and amplifies throughout the trajectory.

**Table 5:** Logistic regression results for complete school trajectory—version with compared social classes.

**Dimension**	**Variable**	**Estimate**	**Std. error**	***z* value**	**Pr(>|z|)**	**Significance**	**Odds ratios**
	(Intercept)	3.42	0.16	21.27	0.000		
Residential and school segregation variables	Residential and school segregation of the elite	0.14	0.06	2.45	0.014	^*^	1.15
	Residential and school segregation of the upper-ascending class	−0.17	0.07	−2.54	0.011	^*^	0.84
	Residential and school segregation of the middle class (aggregated)	0.07	0.04	1.59	0.112		1.07
	Residential and school segregation of the working class (aggregated)	−0.27	0.05	−5.37	0.000	^***^	0.77
Social class variables	Upper-ascending class (vs. elite)	−0.37	0.16	−2.29	0.022	^*^	0.69
	Established middle class (vs. elite)	−0.69	0.16	−4.39	0.000	^***^	0.50
	Emerging middle class (vs. elite)	−1.25	0.16	−7.92	0.000	^***^	0.29
	Consolidated working class (vs. elite)	−2.07	0.16	−13.15	0.000	^***^	0.13
	Precariat (vs. elite)	−3.12	0.16	−19.70	0.000	^***^	0.04
Educational field variables at territorial level	Bilingualism	0.04	0.04	1.13	0.258		1.04
	Full-day schooling	−0.00	0.04	−0.06	0.953		1.00
	Enrollment in public schools	−0.05	0.06	−0.88	0.377		0.95
	Unavailability of school supply	−0.04	0.04	−1.22	0.221		0.96
	Low quality schools	0.10	0.05	2.07	0.039	^*^	1.11
	School failure	−0.07	0.05	−1.49	0.136		0.93
Individual and demographic variables	Sex	0.46	0.01	76.66	-	^***^	1.58
	Belonging to vulnerable population	−0.28	0.01	−28.34	0.000	^***^	0.75
	Lives with both parents	0.68	0.01	91.33	-	^***^	1.98
	Number of people in household	−0.18	0.00	−62.66	-	^***^	0.84
	**Marginal *R*^2^**	**0.24**					
	**Conditional *R*^2^**	**0.26**					
	**Delta_G**	**0.08**					
	**Overall accuracy**	**0.86**					
	**ICC**	**0.16**					

Signif. codes: 0 “^***^” 0.001 “^**^” 0.01 “^*^” 0.05 “.” 0.1 “ ” 1.

Source: Author's calculations based on the methodology described. Bold rows indicate overall model fit statistics for the complete model.

Three transversal patterns emerge from the analysis. First, social class operates as the most robust predictor of educational success. The three types of capital demonstrate significant effects at practically all levels. Cultural capital constitutes the most determinant factor, though its specific configuration varies embodied capital (practices) dominates in primary education (OR = 4.18) decreasing subsequently, while institutionalized capital (family education) increases progressively until upper secondary (OR = 1.61). Literacy emerges as critical factor in graduation (OR = 2.50). Economic capital shows cumulative incidence through durable goods possession (OR = 0.93–1.47), housing (OR = 0.96–1.25), and income particularly in preschool and graduation. Social capital operates primarily through residential rootedness, with effects decreasing from primary (OR = 1.65) to graduation (OR = 1.11).

Second, territory amplifies inequalities through spatial segregation. Residing in Elite zones systematically increases probabilities: +34% primary and secondary, +21% graduation. This effect persists controlling individual capitals, confirming that territory concentrates advantages through educational infrastructure, quality school provision, and peer effects. Educational field variables show differentiated effects: full-day schooling protects in preschool (OR = 1.09), public provision affects primary-secondary, and bilingual schools benefit graduation (OR = 1.11). Local school failure consistently reduces probabilities (OR = 0.79–0.92).

Third, individual variables confirm additional structural inequalities. Females present advantages that amplify at higher levels, as does living with both parents. Conversely, probabilities of successful trajectories reduce in households with vulnerable populations, greater number of members, and single-parent structure.

## 4 Discussion

The results of the study confirm the hypotheses regarding the effects of social class and the combined effects of residential and school segregation on educational trajectories in metropolitan Bogotá. Access to, persistence in, and completion of preschool, primary, and secondary education are strongly mediated by families' socioeconomic position and by the urban and school contexts in which children and youth are embedded. Overall, the findings reaffirm Bogotá's class-based stratification, where educational inequality both reflects and intensifies residential segregation. Territorial disparities and class position interact to reproduce social exclusion. This section discusses the main findings considering the comparative literature, identifying convergences, specificities of the Bogotá case, and implications for contemporary patterns of stratification in highly unequal Latin American contexts.

### 4.1 Confirmation of hypothesis 1: capitals effects across the educational trajectory

The first hypothesis-individuals from lower social classes, characterized by lower volumes of family-level economic, cultural, and social capital, have lower probabilities of accessing, persisting in, and completing education-receives strong empirical support across all five educational transitions. Social class, operationalized through multidimensional capital measures, operates as the strongest structural determinant of educational trajectories in metropolitan Bogotá, surpassing individual-level variables such as gender or family configuration. This aligns with Bourdieu's theory (1986, 2011) emphasizing intergenerational capital transmission and the ongoing relevance of class in ostensibly meritocratic societies (Lareau, 2011; Savage et al., 2013).

All three capital forms exhibit significant and differentiated effects, confirming that educational inequalities arise from multidimensional capital configurations rather than single dimensions. The magnitude proves substantial: precariat individuals show 96% lower graduation probabilities compared to elite counterparts, even controlling for individual characteristics and territorial contexts. Cultural capital demonstrates the most consistent influence, with embodied capital (cultural practices) crucial in primary education (OR = 4.18) and institutionalized capital (parental education) gaining salience at graduation (OR = 1.61). Economic capital effects concentrate at preschool access and graduation-stages where universalization remains incomplete. Social capital operates primarily through residential rootedness rather than formal organizational participation, suggesting that informal territorial networks prove more consequential than formal associational life in contexts of high inequality and limited state provision. These patterns confirm H1 while revealing stage-specific mechanisms through which capitals operate.

### 4.2 Confirmation of hypothesis 2: spatial segregation as educational inequality amplifier

The second hypothesis-that living in residentially segregated areas with concentrated socioeconomic disadvantage and attending schools in contexts of high socioeconomic segregation negatively affects educational probabilities-receives robust empirical confirmation. Territorial segregation operates as a social closure mechanism amplifying class-based inequalities beyond individual family resources. Living in elite-segregated areas increases successful trajectory probabilities by 21–34% depending on level, while working-class segregation reduces these chances proportionally. These effects persist after controlling individual capital endowments, confirming independent contextual effects.

Bogotá exhibits a “double segregation” pattern wherein residential and school segregation compound to structure unequal opportunities. School segregation exceeds residential segregation for upper classes, the emerging middle class, and the precariat-reflecting deliberate strategies to maintain social distance despite territorial proximity (Van Ham et al., 2021). Conversely, for the consolidated working class, residential segregation surpasses school segregation. Educational-field variables show stage-specific patterns: full-day schooling protects at preschool (OR = 1.09–1.80), bilingual school concentration increases graduation rates (+11%), while residing in areas with high school-failure rates reduces completion probabilities (−8% to −21%), consistent with neighborhood-effect and peer-composition literature (Wilson, 1987). These patterns confirm that H2 operates through multiple spatial mechanisms varying across educational stages and class positions.

### 4.3 Specification of capital effects (H1)

#### 4.3.1 The strong influence of cultural capital

The importance of cultural capital across access, persistence, and graduation confirms the Bourdieu's theory (2009, 2020) and more recent updates documenting its persistent relevance in contemporary societies (Andersen and Jæger, 2015; Gorard and See, 2013; Jæger, 2011). However, the differentiated role of embodied cultural capital (crucial in primary school with OR = 4.18) vs. recognized cultural capital (important at graduation with OR = 1.61) complexifies this literature and converges with Sullivan (2001), who documented heterogeneous effects of different cultural-capital forms. The findings suggest that everyday cultural practices in the home (reading, use of technology, cultural activities) are most consequential in early stages (Lareau, 2011), while parental education functions as an anchor sustaining progression through upper levels. Particularly notable is the strong effect of household literacy at graduation (OR = 2.50), confirming that basic cultural capital thresholds remain structural requirements for educational completion in contexts where barriers for working-class families persist.

#### 4.3.2 Economic capital: differentiated effects

Economic-capital variables show slightly weaker effects than cultural capital but with clear internal differentiation. These findings are consistent with evidence on the importance of family economic resources for educational outcomes (Blanden and Gregg, 2004). Income displays distinctive effects at preschool and graduation, possibly due to the lower universality of these levels. The role of income in early access mirrors Magnuson and Waldfogel (2016), who show that higher-income families access early-childhood education more readily. The increasing effect of household assets in later stages (OR = 1.47 in graduation) suggests that economic capital acts not only as an initial facilitator but also as a buffer against dropout at higher levels. Housing conditions also matter, consistent with Von Simson and Umblijs (2021), who show that students in owned housing have higher completion probabilities than those in rental housing—reflecting residential stability and consolidated family resources that help protect educational trajectories.

#### 4.3.3 Residential rootedness as a key component of social capital

The predominance of residential rootedness over organizational participation is particularly revealing in the Latin American context. While being born in Bogotá or having lived there for more than 5 years has significant effects (especially in primary school with OR = 1.65), participation in social organizations has null or negative effects. This contrasts with European evidence where formal social capital predicts educational success (Putnam, 2000). Rootedness suggests that in settings of high inequality and limited state provision, informal networks based on territorial permanence and local ties are more relevant than formal associational life, consistent with findings on migrant populations who show lower probabilities of educational success (Bonal et al., 2019; OECD, 2019). Parental occupation maintains significant, albeit smaller, effects, consistent with neo-Weberian research highlighting the ongoing relevance of occupational position in social-class formation (Goldthorpe, 1993; Bukodi and Goldthorpe, 2013).

### 4.4 Specification of segregation effects (H2)

#### 4.4.1 Territorial segregation as a mechanism of social closure

Living in areas with high concentrations of elites increases the probability of successful educational trajectories by 21–34% depending on the level, while segregation among working-class sectors reduces these chances—patterns consistent with European urban contexts. These findings align with evidence on neighborhood and compositional effects in the U.S. and Europe (Duncan et al., 1997; Galster et al., 2010; Helbig, 2010; Palardy, 2013; Porcel, 2020; Nel·lo, 2021). In Bogotá – where both residential and school segregation are pronounced—the magnitude observed is high, consistent with Latin American contexts where unequal urban development and limited high-quality public goods heighten social-closure mechanisms. However, direct causal comparison between regions remains limited and requires harmonized designs.

#### 4.4.2 Variation in educational-field effects across trajectory stages

The effects of educational-field variables show clear stage-specific patterns. Full-day schooling protects only at preschool (OR = 1.09 in multilevel and OR = 1.80 in logistic models), aligning with Cooper et al. (2010), who found that full-time schedules benefit early education but fade later—suggesting that it functions more as early-childhood care provision. Living in areas with high concentrations of bilingual schools increases graduation rates (+11%), reflecting their role as territorial markers of status in unequal urban environments, where elite schools cluster in specific neighborhoods (De Mejía, 2002; Oberti and Jacobs, 2007). The counterintuitive protective effect of low-quality school zones reflects the coverage—quality trade-off observed in Latin American educational expansion (Reimers, 2000): areas with lower-performing schools often have more available places, easing reentry and continuity. Finally, living in areas with high school-failure rates reduces the probability of completion (−8% to −21%), consistent with neighborhood-effect and peer-composition literature (Wilson, 1987), and in Latin America with evidence that residential and school segregation amplify dropout risks.

### 4.5 Comparative context

#### 4.5.1 Patterns of educational stratification in Latin America

The Bogotá findings reflect broader Latin American patterns characterized by high educational segmentation, strong socio-economic school segregation, and persistent effects of class on achievement despite major educational-coverage expansions (Bonal and Bellei, 2019). The magnitude of the observed disparities—such as a 96% lower graduation probability for the precariat compared to the elite—aligns with some of the highest levels of educational inequality documented in the region. Notably, school segregation often exceeds residential segregation among upper-class groups, reflecting deliberate elite strategies to maintain social distance even when sharing territories with other classes—clearly documented in Santiago (Valenzuela et al., 2013) and Lima (Reátegui et al., 2022). Similar patterns of school choice and segregation strategies have been identified in Mexico City (Blanco et al., 2014), though the comparative magnitude requires further research. These findings build on previous research that has examined residential segregation and its effects on disadvantaged groups in Latin American cities, including Santiago (Sabatini, 2006), Montevideo (Kaztman and Retamoso, 2007), Buenos Aires (Salvia, 2007; Serrati, 2023), and in European contexts such as Barcelona and Madrid (Nel·lo, 2021) or European capital cities (Tammaru et al., 2016).

### 4.6 Theoretical implications: beyond Bourdieu in contexts of extreme inequality

These findings both validate and extend Bourdieusian class theory in three theoretically consequential ways. First, the stage-specific effects of capital forms complicate the habitus concept: if embodied capital operates most powerfully in early childhood (OR = 4.18) while institutionalized capital dominates completion (OR = 1.61), class reproduction mechanisms are not uniformly “durable and transposable” but contingent on educational stage structure. Second, the double segregation pattern—wherein school segregation exceeds residential segregation for upper classes (93% vs. 86%) but reverses for working classes (41% vs. 61%)—suggests class-differentiated spatial strategies: elites actively construct social distance through school choice even when residentially proximate to other classes, while working classes experience involuntary residential concentration. Third, territorial context accounts for 6–23% of variance (highest in secondary), a magnitude larger than typically observed in European studies (8–15%), suggesting segregation mechanisms intensify in contexts of extreme inequality where state provision weakens. These patterns indicate that Bourdieusian theory, developed in relatively egalitarian postwar France, requires refinement for Latin American contexts where weak institutional mediation allows class effects to operate more directly and territorially.

## 5 Limitations and future directions

This study presents several limitations that open relevant avenues for future research. First, the analysis is cross-sectional and reconstructs cohorts retrospectively, limiting causal inference on capital accumulation over time. Longitudinal designs following students from preschool to upper-secondary education would allow more precise identification of these mechanisms and permit disentangling contemporaneous from cumulative effects of family resources and territorial contexts.

Second, potentially omitted variables merit consideration: (1) educational aspirations and family expectations, (2) cognitive and non-cognitive skills, and (3) parental educational practices. However, the literature suggests these factors are deeply determined by social class and function primarily as transmission mechanisms rather than independent variables. Cunha and Heckman (2008) and Heckman and Kautz (2012) demonstrate that skill gaps between socioeconomic groups emerge early and amplify through differentiated parental investments, while Lareau (2011) shows that parental practices are deeply stratified by class, questioning their treatment as exogenous. The relatively high marginal and conditional *R*^2^ values in our multilevel models suggest that multidimensional capital measures capture much of how these factors operate. Nevertheless, without direct measurement, residual endogeneity cannot be completely ruled out. Future quasi-experimental designs incorporating direct measures would enable more rigorous causal evaluation.

Third, although multilevel models capture territorial effects, they do not uncover the specific mechanisms through which urban environments influence educational trajectories. Qualitative or mixed-methods studies examining institutional resources, peer networks, and neighborhood-level social capital could deepen this understanding. Fourth, the focus on metropolitan Bogotá limits generalizability to other Latin American cities with different urban configurations, school systems, and stratification regimes. Finally, the analysis period includes COVID-19 years, whose effects on childcare and preschool access require further research to differentiate structural patterns from temporary disruptions.

## 6 Conclusions

Since mid-twentieth century, education has emerged in Latin America as a promissory vehicle for social mobility and distributive justice. Nevertheless, in metropolitan Bogotá—as in other regional metropolises—this promise has remained unfulfilled: despite advances in universal coverage, the educational system continues operating as a site of social hierarchy reproduction, wherein social class of origin, residential segregation, and educational field segmentation interweave to structure profoundly unequal educational trajectories.

### 6.1 Social classes from a relational and multidimensional approach

Multidimensional analysis of economic, cultural, and social capital identifies six social classes exhibiting radically differentiated educational trajectories: Elite (0.2%), Upwardly Mobile Upper Class (2.6%), Established Middle Class (33.5%), Emerging Middle Class (6.5%), Consolidated Working Class (55.2%), and Precariat (2.1%). High school graduation gaps evidence persistent educational hierarchies: whereas 100% of Elite and upper-class students complete upper secondary education, merely 85% of the Consolidated Working Class and 75% of the Precariat achieve completion. This inequality derives from differential capital possession that structures unequal capacities from class origin, reproducing intergenerational stratification patterns characteristic of Latin American societies.

### 6.2 Empirical validation of reproduction mechanisms

Findings confirm the determinant incidence of social class on educational trajectories through three validated structural mechanisms: (a) unequal capital concentration according to hierarchical social position, (b) residential segregation determining differentiated access to urban and educational resources, and (c) school segregation concentrating same-class students in homogeneous establishments, thereby limiting exposure to social diversity. Controlling for all variables, Precariat membership vs. Elite reduces high school graduation probability by 96%. Social class thus determines not merely the starting point but accumulates effects throughout the trajectory: gaps widen at each educational transition, evidencing that the educational system magnifies rather than compensates for inequalities of origin. Social class thereby emerges as the strongest predictor of educational trajectory, surpassing gender or other variable effects.

### 6.3 Urban fragmentation and residential segregation

Bogotá exhibits a “U-shaped” socio-spatial segregation pattern characteristic of Latin American cities with elevated inequality levels, wherein extreme classes demonstrate maximum isolation: the Elite manifests extreme segregation (93% at census block, 86% at neighborhood level) concentrated in the north, while working classes predominate in the southern periphery. This hierarchical fragmentation determines territorially variable educational opportunities, consolidating a system wherein residential location—itself determined by social class—strongly predicts educational trajectory quality.

### 6.4 Segmented educational field and school segregation

The educational field is structured through hierarchical circuits with pronounced public/private division and quality, schedule, and bilingualism differentials, constituting an educational stratification system that reproduces and amplifies social hierarchies. School segregation approaches absolute levels among upper classes: 99% attend selective private schools with monthly investments of USD $800-1,200, full-day schedules (>95%), and bilingual instruction (33–67%), whereas working classes attend predominantly proximate public schools (74–88% reside nearby) of medium-to-low quality. For Elite and Precariat, school segregation exceeds residential segregation, evidencing complete separation and null interaction between upper classes and society's remainder.

### 6.5 Class capitals and inequality reproduction

Individuals possessing lower capital systematically demonstrate reduced probability of completing and graduating from high school. Cultural capital emerges as the most determinant factor, particularly embodied practices during early stages and household educational credentials in final stages. Economic capital exhibits consistent incidence, with durable goods possession, housing conditions, and income standing out. Regarding social capital, residential rootedness proves most consequential, followed by household heads' occupation. “Neighborhood effects” operate differentially: residing in Elite concentration zones increases graduation probability by 21–44% depending on educational stage, whereas residing in working-class zones systematically reduces it, evidencing that territory functions as a social closure mechanism concentrating advantages among upper classes while constraining working-class trajectories.

### 6.6 Stratification patterns in Latin American context

This study evidence contemporary stratification patterns in a Latin American metropolis wherein, notwithstanding educational access universalization, social hierarchies persist and reproduce through double residential-school segregation. Social class constitutes an articulating axis of multiple inequalities that mutually reinforce across urban space and the educational field, consolidating an intergenerational reproduction system of privileges and disadvantages. Findings suggest that universal educational policies ignoring class structure, educational inequality/segregation, and spatial segregation possess limited compensatory potential in high social inequality contexts, necessitating structural measures simultaneously addressing territorial planning, public education strengthening, and social integration. The Bogotá case suggests these class-structured reproduction patterns may characterize other Latin American metropolises, raising questions regarding social hierarchy persistence across the region despite decades of educational expansion and opportunity universalization efforts.

## Acknowledgments

This article is based on research conducted as part of the author's Master's thesis in Economics and Politics of Education at Universidad Externado de Colombia. The author gratefully acknowledges Santiago Téllez Rojas for academic supervision and guidance throughout the research process, and María José Álvarez Rivadulla and Óscar Alfonso for valuable feedback as thesis committee members. The author thanks the Colombian National Administrative Department of Statistics (DANE) for census and household survey data; the Ministry of National Education (MEN) for educational administrative records; the Colombian Institute for Educational Evaluation (ICFES) for standardized test results; and the Bogotá District Education Secretariat (SED) for additional educational data. All data sources are publicly available for research purposes.

## Data Availability

Publicly available datasets were analyzed in this study. This data can be found at: The datasets analyzed in this study are publicly available through the official repositories of the Departamento Administrativo Nacional de Estadística (DANE) and the open data platforms of Bogotá District and the Ministry of Education of Colombia. 2018 National Population and Housing Census (CNPV-2018) Repository: DANE – Microdata Catalog Direct link: https://microdatos.dane.gov.co/index.php/catalog/643 (Statistical operation ID: CNPV 2018). 2021 Multipurpose Household Survey (EM-2021) Repository: DANE – Microdata Catalog Direct link: https://www.dane.gov.co/index.php/estadisticas-por-tema/pobreza-y-condiciones-de-vida/encuesta-multiproposito (Statistical operation ID: EM 2021). Territorial cartography (UPZ, sections, blocks, census sectors) Repository: DANE – Geostatistical Framework/Divipola Direct link: https://geoportal.dane.gov.co/geovisores/sociedad/cnpv-2018/ (Geostatistical framework: seccion, manzana, sector). Bogotá District Open Data (educational supply, school locations) Repository: Portal de Datos Abiertos – Bogotá Direct link: https://datosabiertos.bogota.gov.co. Ministry of Education of Colombia – Educational institutional data Repository: Datos Abiertos – Ministerio de Educación Nacional Direct link: https://www.mineducacion.gov.co/portal/Ministerio/Informacion-Institucional/349303:Datos-Abiertos (School directory/SIMAT institutional information). These datasets are fully public and require no request or authorization. DANE does not use accession numbers; instead, each dataset has an official catalog ID, which I included above. None of the original datasets are included in your Supplementary material (only derived indicators), which is correct.

## Footnotes

1 UPZs (Unidades de Planeamiento Zonal) are intermediate administrative units in Bogotá that group approximately 8 – 15 neighborhoods with similar socioeconomic and morphological characteristics. Their territorial extension ranges between 2 and 15 km^2^ (typically 5 – 10 km^2^), and they contain populations of 50,000 – 80,000 inhabitants. UPZs constitute the spatial level between the locality and the neighborhood.
